# A Novel Real-Time MATLAB/Simulink/LEGO EV3 Platform for Academic Use in Robotics and Computer Science †

**DOI:** 10.3390/s21031006

**Published:** 2021-02-02

**Authors:** Nicolas Montes, Nuria Rosillo, Marta C. Mora, Lucia Hilario

**Affiliations:** 1 Department of Mathematics, Physics and technological sciences, University CEU Cardenal Herrera, C/San Bartolome 55, CP 46115 Alfara del Patriarca (Valencia), Spain; nrosillo@uchceu.es (N.R.); luciah@uchceu.es (L.H.); 2 Department of Mechanical Engineering and Construction, Universitat Jaume I, Avda de Vicent Sos Baynat s/n, CP 12071, Castellon, Spain; mmora@uji.es

**Keywords:** STEAM, LEGO Mindstorms, EV3, MATLAB, Simulink, computer science, robotics, computational thinking

## Abstract

Over the last years, mobile robot platforms are having a key role in education worldwide. Among others, LEGO Robots and MATLAB/Simulink are being used mainly in universities to improve the teaching experience. Most LEGO systems used in the literature are based on NXT, as the EV3 version is relatively recent. In contrast to the previous versions, the EV3 allows the development of real-time applications for teaching a wide variety of subjects as well as conducting research experiments. The goal of the research presented in this paper was to develop and validate a novel real-time educational platform based on the MATLAB/Simulink package and the LEGO EV3 brick for academic use in the fields of robotics and computer science. The proposed framework is tested here in different university teaching situations and several case studies are presented in the form of interactive projects developed by students. Without loss of generality, the platform is used for testing different robot path planning algorithms. Classical algorithms like rapidly-exploring random trees or artificial potential fields, developed by robotics researchers, are tested by bachelor students, since the code is freely available on the Internet. Furthermore, recent path planning algorithms developed by the authors are also tested in the platform with the aim of detecting the limits of its applicability. The restrictions and advantages of the proposed platform are discussed in order to enlighten future educational applications.

## 1. Introduction

In the last few years, low-cost hardware/software systems are having a very important role in different levels of education worldwide. Many public and private educational institutions around the world, from elementary schools to universities, are using them to improve the teaching experience in very different subjects and specially those offering technological degrees. The most commonly used low-cost development devices are typically: Arduino, Raspberry Pi, and Kinect. In the case of robotics, a variety of low-cost mobile robots have appeared in the last few years, such as Adept, E-Puck, Moway, LEGO Mindstorms, etc. [1]. These devices do not offer the same accuracy as industrial robots but enough for educational purposes in engineering or even experiments and research tests. In this sense, the contribution of robotics and computational thinking to education has become a research topic [2].

Although every low-cost platform is useful, LEGO Mindstorms has played a striking role in college engineering education in the last decade. The fundamental reason is that LEGO Mindstorms series of kits include software and hardware to build customizable and programmable robots [3]. They include an intelligent brick, which is a computer to control the system, a set of modular sensors and motors, as well as LEGO parts from the technical line to build the mechanical systems, allowing the creation of many different robotic systems. LEGO robotics is fundamentally a constructivist tool, where students leverage their knowledge and experience to solve real-world problems and to consistently question and challenge that knowledge as they develop their solution [4]. There are three types of bricks to control LEGO robots: Robotic Command Explorer (RCX), The Next Generation (NXT, available since 2006), and the The Evolution, 3rd Generation (EV3, available since January 2013). The system clock speed in the EV3 increases to 300 MHz, six times faster than that of the NXT. One of the main new features of the EV3 over the NXT and the RCX is the introduction of a Wi-Fi connection to a network, which opens the possibility of communicating with the environment and, therefore, a new range of interactive applications can be envisioned.

**Figure 1:**
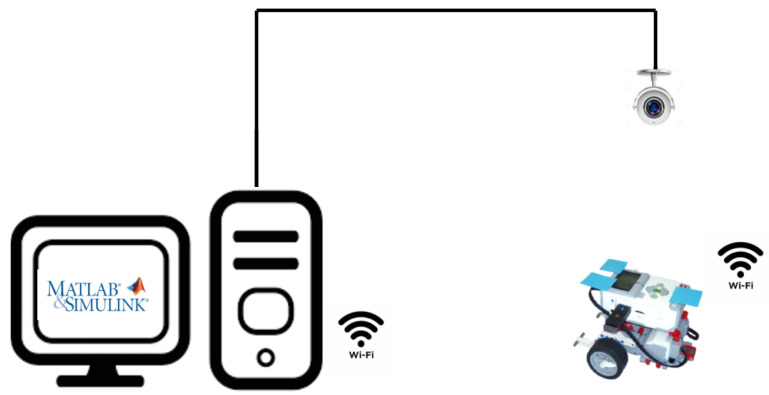
Experimental setup to test the platform.

LEGO robots are frequently used in the teaching activities of many subjects or disciplines. These activities often combine a LEGO robot with different programming languages and platforms. In the university and research fields, the most used platforms with these robots are MATLAB and Simulink. Both programming environments provide libraries to work with LEGO robots. In MATLAB, the robot is considered a device to be teleoperated while in Simulink a program can be uploaded into the robot as well as its behavior monitored, taking into account the restrictions imposed by the robot’s hardware and software. In our previous work [5], a combination of MATLAB and Simulink was proposed to develop complex and real-time applications with a LEGO EV3 robot. In that work, Simulink communicated with the LEGO robot while MATLAB and Simulink were running in parallel on the PC and exchanging information between them, as shown in Figure 1, offering a new possibility to work with the three platforms.

The objective of the present work is to test and evaluate the MATLAB/Simulink/LEGO EV3 platform in order to explore its ability to act a generic platform in engineering education. For this purpose, three research questions are posed and assessed through a Project Based Learning (PBL) methodology applied to different students and profiles (computer science an robotics) of several educational levels (undergraduate, graduate and PhD).

The paper is organized as follows. Section 2 provides a state of the art of the use of LEGO robots in education, from early childhood education to doctoral studies as well as their use in research. Section 3 details the purpose of the research presented in the paper. Section 4 presents the methodology of this work, where the aforementioned educational platform based on the LEGO EV3 system is described. Section 5 details the results of the use of the platform in different educational environments with the aim of testing the platform capacities. Advantages and disadvantages of the platform are indicated in Section 6. Finally, conclusions and future works are drawn in Section 7.

## 2. LEGO Mindstorms: From Kids to Researchers

LEGO robots have been used for curricular and extracurricular school activities related to technology. A literature review is presented in the following subsections.

### 2.1. LEGO for Kids

One of the most important activities carried out for kids between 9 and 14 years old is the First LEGO League [6], a robotics competition created by the LEGO Group in 1999 aimed at building and programming robots that perform different tasks. Each year a new challenge is raised with various missions to be completed by the robot. The robot runs on a mat and the team must design and program the robot to score as many points as possible in a period of time of two and a half minutes. The FLL is a good way to foster children’s interest towards the disciplines embraced by the STEM education: Science, Technology, Engineering, and Mathematics, [7]. Moreover, LEGO robots have been proven helpful in the teaching of subjects and skills at school such as mathematical and computational thinking [2]. For instance, in [8,9] the effect of LRT (LEGO Robotics Training) on learners performance in mathematics is investigated. In [10,11], a LEGO NXT and EV3 were respectively used as teaching elements in order to motivate the generation process of computational thinking in students of different school levels. In high educational levels, numerous initiatives have been carried out focused on engaging teenagers in the computer science knowledge through LEGO robots. In this sense, mentoring programs are one of the key strategies used by universities to attract students, as in [12] with the organization of a service-learning robotics outreach and mentorship program in an engineering department of a developing country by means of various robotic platforms such as LEGO Mindstorm NXT and EV3.

### 2.2. LEGO for Undergraduate and Postgraduate Students

There are many types of LEGO Mindstorms applications in engineering teaching for undergraduate and postgraduate students in the literature. The next subsections summarize the most relevant ones.

#### 2.2.1. Teaching Computational Thinking and Programming

A recent research [13] has shown the positive influence of the introduction of the robotics technology in the engineering students’ achievements when learning computer programming algorithm logics. The statistical results show an increase in the student performance when LEGO robots are employed in introductory computer programming courses. In fact, LEGO Mindstorms have been used to teach the most common programming languages, such as C with its variants (ANSI-C [14,15], Robot-C [16,17], or NXC [18], Java (JADE-LeJOS) [19,20], JS-Eden [21,22], ADA ([23,24]), Phyton [25], Labview [26,27]), and MATLAB/Simulink. Among them, MATLAB is the most used programming language with LEGO Mindstorms [28,29,30,31,32,33]. Although it is not a free software, MATLAB is widely used in universities due to the student licenses and to the free package developed by MathWorks for MATLAB/Simulink called “Simulink Support Package for LEGO MINDSTORMS”, which includes Simulink blocks for LEGO robots in order to program their behavior. The Simulink tool is a graphical interface which makes the programming task very visual and simple.

#### 2.2.2. Teaching Control Techniques

LEGO Mindstorms NXT has been extensively used to address basic control theories and concepts from a practical perspective. The work presented in [28] introduces this system in three laboratory sessions of a standard third-year undergraduate course to demonstrate control techniques and theories like system modeling, PID, state feedback control, and estimator design. It is also used in [29] to explain stability, steady state error, as well as data acquisition from a variety of sensors to undergraduate students.

Advanced control techniques have been also taught to undergraduate students by means of LEGO Mindstorms NXT: modeling and control of step motors as well as Kalman filter and kinematic control in [17]. In [34], a LEGO Mindstorms NXT robot connected to MATLAB/Simulink is used to explain control systems. In particular, the design of a PID controller is developed to achieve the line tracking of the mobile robot. Along the same lines, the multi-variable controller design of a LEGO Mind-storms NXT robotic arm is presented in [35] for educational purposes, which is a valuable model for learning multivariable controller design for undergraduate students. An artificial neural network-based controller is presented in [20] that is capable of behaving meaningfully in a LEGO sumo wrestling context (LEGO NXT robot). The goal is to offer useful practical guidance to allow performing evolutionary experiments with not many time-consuming trial-and-error phases.

#### 2.2.3. Teaching Robotics

Similarly, robotics foundations as well as particular robotics applications have been taught to students by means of LEGO Mindstorms robots in every level of university education. Many applications have been carried out in undergraduate robotics courses, mainly with the LEGO Mindstorms NXT version. For instance, a set of projects are elaborated in [18] that develop intelligent robotic systems in a computer science degree, implementing reactive and deliberative agents, rule-based systems, graph-search algorithms, and planning methods. Robot control techniques are taught to senior undergraduate engineering students in [26] by means of an NXT system, including basic control, tele-operation, line tracking, and fuzzy logic techniques as well as reactive navigation. An educational hands-on project is proposed in [31] for learning guidance and control. In this project, a LEGO Mindstorms NXT together with a web camera and tractable tools are used for searching and mapping an obstacle in an indoor environment. LEGO Mindstorms NXT has also been employed at graduate level, as in [19] where it is used to introduce students to the concept of localization in mobile robots using the extended Kalman filter in the fifth year of an integrated master’s program. Or in [36] where LEGO Mindstorms NXT is used in laboratory practices with manipulators in a M.Sc. degree in Computer and Mechatronic Engineering. Different models of manipulators are constructed: an anthropomorphic and Scara robot and even a Cartesian robot able to write. Complex robotics systems have been developed and used as transversal platforms for undergraduate and graduate levels. In [33], a Mechatronic Demonstrator was proposed using the LEGO NXT-2 Platform. It was a humanoid robot suitable for the study of the key elements of mechatronic systems: mechanical systems, sensors, computer interfacing, dynamics, and application development.

In [32], a LEGO Mindstorms NXT ballbot is employed to teach linear controllers with parameter variations. The ballbot dynamics is based on the spherical inverted pendulum. The inverted pendulum is also considered as at-size scenario in [30] to outline both the theoretical and practical aspects of the Model Predictive Control theory. Finally, LEGO Mindstorms is used in [36,37] to develop a self-stabilized bicycle for teaching control design, as its complex control is also based on the inverted pendulum.

#### 2.2.4. Teaching Machine Learning and Artificial Intelligence

Advanced concepts and techniques related to Machine Learning and Artificial Intelligence have also been taught through LEGO Mindstorms. A 12-years teaching experience on Artificial Intelligence is presented in [16] using LEGO RCX, NXT, and EV3 Robotics platforms for both undergraduate and graduate classes at Western Washington University. In [21], LEGO Mindstorms EV3 robots are used with a novel Open Learning Environment for Artificial Intelligence (OLE-AI) to teach reinforcement learning and artificial neural networks (ANNs) concepts to computer science students. More recently in [22], LEGO EV3 robots and MATLAB AB are used to teach optimization techniques and, specifically, the principles of classical and metaheuristic optimization algorithms at the undergraduate level.

### 2.3. LEGO Mindstorms for Research

The possibilities of LEGO Mindstorms along with its low cost have encouraged its use also in experimental research.

In [38], the potential of LEGO EV3 mobile robots in industry is explored, especially as alternatives to human operators in life-threatening professions. A snail robot is constructed based on LEGO Mindstorms EV3 that is capable of climbing up a ladder.

In [39], an anthropomorphic phantom is built using LEGO Mindstorms EV3 for Adaptive Radiation Therapy (ART) purposes in order to simulate the breathing processes in the lung district during treatments. The humanoid phantom is tested in clinical practice, where breaths and CT studies of 12 patients are analyzed. By using LEGO, it is possible to reproduce the actual patients’ conditions and simulate normal and even abnormal behavior during the course of therapy, allowing spatial motion estimation.

The Second Order Sliding Mode Controller (SOSMC) using the Super Twisting algorithm for the two-wheeled inverted pendulum (TWIP) considering the dynamic mathematical model of the LEGO EV3 TWIP is presented in [40]. The contribution of this paper is the real-time implementation of the Super Twisting SOSMC algorithm to balance the LEGO TWIP in an upright position. Recently, the LEGO Mindstorms NXT robotics has been presented in [41] as the core component of a low-cost multidisciplinary platform for assisting elderly and visually impaired people. It facilitates, without any special training and at low cost, the use of such device for interpersonal communication and for handling multiple tasks required for elderly and visually impaired people in-need. The research project provides a model for a large-scale implementation, tackling the issues of creating additional functions in order to assist people in-need. More recently in [42], a two-wheeled LEGO EV3 robot is used to test a new controller. In this case, a fuzzy logic controller is implemented to stabilize the robot with a Particle Swarm optimization algorithm for the optimum performance of the system.

## 3. Research Objectives

The previous literature review shows the big amount of work done using the LEGO NXT platform at all educational levels, including STEAM subjects (Science, Technology, Engineering, Arts, and Maths), Computer Science, and Robotics subjects as well as research. However, the NXT version of the LEGO systems does not allow for real-time performance, which prevents the development of interactive teaching/learning methodologies with students, as well as the implementation of complex algorithms (RT programming based on threads). Conversely, the more recent EV3 version has barely been used even though it shows a higher computational capacity compared to the NXT version as well as real-time performance. Its advantages have been tested and presented in [43].

The present paper extends the work initiated in [5], where the authors presented a MATLAB/Simulink/LEGO EV3 platform for teaching robotics subjects in engineering degrees. In the current work, this educational tool has been extended to the teaching of computer science topics as well as research. In this sense, this platform allows the development of a real-time framework able to improve most of the works explained in the previous section. In particular, the present work gives light to the enhanced features of the LEGO EV3 brick and develops its possibilities focused on interactive applications for undergraduate and graduate students as well as research in the fields of robotics and computer science.

The main objective of this work is to test and validate the low-cost real-time MATLAB/Simulink/LEGO EV3 platform and its new capabilities to enhance the teaching of many different subjects based on the most popular software used in engineering degrees, MATLAB/Simulink. In other words, to explore the possibility of using it as a generic platform in engineering education. For this purpose, different interactive projects with specific goals have been developed by students of different levels (last-year undergraduate, graduate and PhD students). The validation implies assessing if building and programming interactive applications with the proposed platform make students integrate the knowledge they already have regarding control, robotics and computer science in a simple way. This knowledge has been divided in:

- Abstract knowledge: Development of mathematical models that emulate the reality. In particular, kinematic and dynamic models that will be used in the software in order to generate the proper robot trajectory.
- Physical and procedural knowledge: Understand and manipulate physical systems such as sensors (camera, encoders), actuators (motors, wheels), mechanical devices (transmissions) and communication systems (Wi-Fi, Bluetooth).
- Logico-mathematical knowledge: Related to the computational thinking required to develop effective programs capable of moving the robots and successfully performing the required tasks.

Three research questions are posed in this work, each one of them related to each particular type of knowledge:

1. How well do students develop an understanding of mathematical models and use them in robot programming with this platform?
2. What level of procedural competence do students acquire thanks to the interaction with robots?
3. Can the use of this platform facilitate the development of computational thinking (CT) skills in an easy way?

The novelty of the proposed educational platform with respect to the existing systems is the possibility of a real-time communication with the environment using MATLAB in a very simple manner. This implies that any external device can provide information to the system during its operation, expanding the possible applications of the system as real-time performance is now possible. In general, the existing Simulink-based platforms are only capable of compiling the program and sending it to the robot but no communication with the environment is possible in real time and, thus, interactive projects cannot be performed. In this work, a robotic navigation application is presented as an example of interactive application where images grabbed by a web camera are processed in MATLAB for obstacle detection and the extracted information is sent to the robot in real time, in order to navigate avoiding the detected and unexpected obstacles.

## 4. Materials and Methods

This section details, first, methodology used in this research work; second, the software used with LEGO EV3 robots, with an example of robot control application; third, the novel educational platform: the software, the hardware and the experimental setup; fourth, the experimental validation carried out.

### 4.1. Methodology

The research presented in this work is based on the well-known educational methodology Project-Based Learning (PBL) [44]. In order to answer the research questions, the PBL methodology has been used in the form of case studies proposed to students of different educational levels, with specific objectives for each interactive project. Specifically, two undergraduate computer science students have developed their final degree project, a graduate student specialized in robotics has developed an internship project and PhD robotics students have developed research tests. In particular, different path planning algorithms have been implemented in an environment with obstacles with a LEGO EV3 robot, with different levels of difficulty. The undergraduate students have implemented and reused previously existing code on the web for classical navigation algorithms (RRT and potential fields), the master’s student has implemented a current and complex navigation algorithm based on parametric curves [45]. Finally, the PhD students have used the platform to develop a new algorithm recently published in [46].

Qualitative empirical data have been collected for the assessment of the research questions. In particular, student reports, field notes and a set of mini-interviews have been gathered. In addition, quantitative empirical data regarding external observation and evaluation have been collected from the score of the three members of the evaluation committee of the final degree and the internship projects of the undergraduate students and the graduate student, respectively. The members belonged to the computer science and automation fields. The evaluation was based on a rubric according to the University regulations that evaluates both transversal and specific competences for this type of projects.

Finally, a retrospective analysis has been carried out with the goal of providing grounded results that could be adapted to other circumstances.

### 4.2. Software Description

MathWorks^®^ has developed two free packages to work with LEGO robots, both from MATLAB^®^ and from Simulink^®^. The first package, “MATLAB Support Package for LEGO MINDSTORMS EV3 Hardware”, allows the measurement and control of sensors and actuators. In this mode, the code execution is performed on the computer and the communication with the robot is carried out through functions that allow to measure and to upload the actions. The second package has been developed for Simulink and is called “Simulink Support Package for LEGO MINDSTORMS”. It allows the programming of LEGO^®^ bricks with Simulink block diagrams. The blocks included in this toolbox are simpler than those available in the LEGO software and are very easy to use, as they work as common Simulink blocks. It is possible to find blocks for modeling sensors (color, gyro, ultrasonic, infrared touch, and ultra-sonic sensors) as well as for controlling motors and reading data from encoders and other sensors. Once the program is finished, Simulink compiles it to C++ and transfers it to the LEGO Mindstorms EV3 brick through the selected connection. Common Simulink blocks can also be used in the LEGO software, with a similar function.

These two packages allow the development of very diverse educational applications, due to the ability of LEGO blocks to transform into almost anything. Depending on the package used, the applications can be classified into Off-Board and On-Board. If the MATLAB package is used, the computational cost falls on the computer but, as an advantage, it is possible to execute any function already developed or programmed in MATLAB code. If the Simulink package is used, the code is compiled to C++ and then transferred to the robot in order to execute it.

In addition, there is a third option regarding the code execution in the LEGO EV3 brick. This option is very interesting and key to the contribution of the present work: the Simulink^®^ External Mode (EM). In the real-time EM, the Simulink^®^ Coder generates an executable file that dynamically links the algorithm code with the I/O driver code generated from the I/O blocks. This file runs in the operating system kernel mode on the host computer, exchanging parameter data with Simulink^®^ by means of a shared memory interface. Thanks to the Simulink^®^ EM, those parameters that change in the Simulink^®^ block diagram are immediately updated in the real-time application. The EM executable file is fully synchronized with the real-time clock. Changes in the parameter values can be carried out during the execution of a real-time application by means of the Simulink Real-Time Explorer or the MATLAB code. In the Simulink^®^ External Mode, it is possible to change the parameters directly in the block or indirectly through MATLAB^®^ variables, creating them as Tunable Global Parameters. These type of parameters must be defined and initialized at the beginning of the application, for them to be considered as global variables.

#### An Example of Robot Control Application

**Figure 2:**
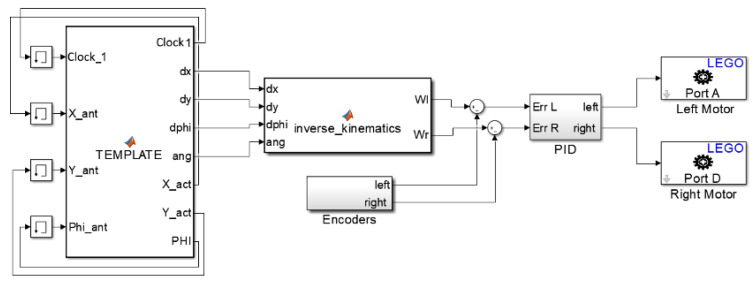
Simulink block diagram of the LEGO EV3 robot control application. (courtesy of Springer Nature [5]).

A robot control application is detailed here in order to explain the properties of the proposed framework. For that purpose, a Simulink^®^ block diagram is first created with the system model, displayed in Figure 2.

The following blocks can be distinguished in the model: Template, PID Controller, Engines (left and right motors), encoders, and inverse kinematics (IK). The robot IK block transforms the robot velocity into wheel velocities. The inputs to the PID Controller block are the errors between the computed wheel velocities from the IK block and the real wheel velocities measured by the encoders. The output is the power to be supplied to the wheels motors. The Template block is a MATLAB function block, where path planning algorithms can be coded to be tested in the LEGO Robot. This block has three types of associated variables: Inputs, Outputs, and Parameters. These variables are defined in the “\Port and Data Manager” window of the MATLAB function. The first two variables are the Inputs and Outputs in the Simulink model but Parameters are related to the MATLAB Workspace. Parameters can be selected as ‘tunable’, meaning that they can be modified in real-time through the MATLAB workspace, or ‘non tunable’ if they are constant and initially set when the Simulink model is compiled. For instance, the position [X1; Y1] of an object 1 can be updated in real-time defining a ‘tunable’ parameter POSITION 1. The definition and initialization of this parameter in the .m le is very simple:

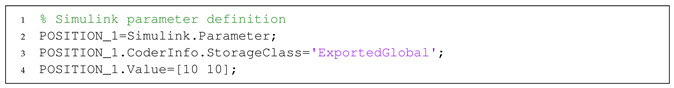



Then, in order to update the variable changes in the application, it is only necessary to indicate it in the code. For the previous example, the update in the object 1 position could be performed as follows, where ‘Block Diagram’ is the name of the Simulink le located in the same folder as the .m file:

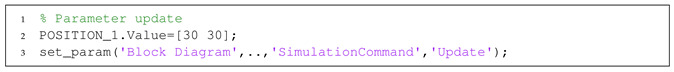



**Figure 3:**
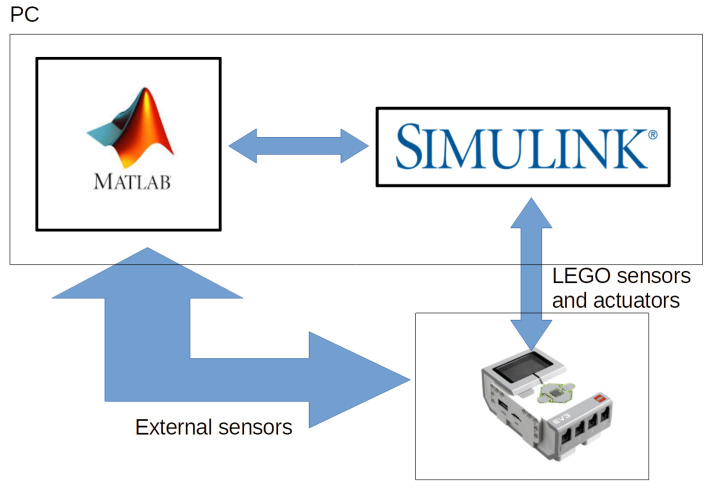
Linking MATLAB-Simulink-EV3.

Parameters must be defined and initialized only once. However, each time a parameter value is changed in MATLAB, the value must be reloaded in Simulink. A diagram of the proposed parameter update system is depicted in Figure 3.

### 4.3. Real-Time Platform for Academic Use

#### 4.3.1. Hardware Setup

The experimental setup described in Figure 1 has been employed in different students’ projects and consists of a 2 × 2 m white square table located on the floor and a web camera located on the ceiling, as shown in Figure 1 (left). The camera is connected to the computer via a USB connection and is used in the MATLAB environment for grabbing and processing images in real time. As a result, obstacles are detected and their locations are sent to the robot for it to avoid them while navigating to the goal.

An omnidirectional LEGO EV3 robot has been built with the MINDSTORMS kit and has been equipped with three blue square labels (see Figure 1) to facilitate the calculation of its position and orientation using the images captured by the camera. A red disk indicates the goal position for path planning purposes. The robot dimensions are included in the Inverse Kinematics Simulink block and the path planning algorithms are programmed in the Template Simulink block, both displayed in Figure 1. The robot configuration (position and orientation), as well as the goal, are defined in MATLAB as ‘tunable’ parameters because they are sent by the computer to the robot after the image processing. To this end, the LEGO robot has been equipped with a USB dongle to communicate with the computer through a Wi-Fi network.

#### 4.3.2. Software Setup

For every project, two different .m files have been developed and loaded in the MATLAB environment. The first one is devoted to the definition and initialization of the parameters and the camera. The second one is in charge of taking snapshots in real-time with the camera, processing them to compute the present position and orientation of the robot and the destination and, finally, updating the parameters in Simulink. In order to switch on the platform, it is mandatory to follow the next sequence:

1. Execute the .m file containing the definition and initialization of the parameters and the camera in the MATLAB environment.
2. Execute the Simulink Template block in External Mode.
3. Execute the .m file that updates the current robot position and orientation as well as the goal configuration.

### 4.4. Experimental Validation

The advantages of the framework proposed in the present paper have been tested by students through the development of interactive projects, following a PBL methodology. In particular, different path planning algorithms have been implemented with a LEGO EV3 robot, which allow the navigation of the robot in an environment with obstacles. In these applications, the robot must reach a specific target starting from an initial random position without colliding neither with the obstacles of the environment nor the environment itself. Three types of interactive projects have been developed by students with different objectives, described in the next paragraphs.

- Type A: Bachelor’s Degree Final Projects
  As mentioned earlier in the introduction, MATLAB is broadly used in universities due to the student licenses provided by Mathworks. In addition, a lot of universities and researchers provide open access to the source code of their algorithms with the aim of validating them by the robotics community. The students were in the last bachelor computer science course and the goal of their Bachelor’s Degree Final Projects was to explore the web to find open source code developed in MATLAB for the most common motion planning algorithms with the aim of implementing them in the LEGO EV3 robot. These students had not previously worked in MATLAB, LEGO or robotics during the degree.
- Type B: Internship Student Project
  The case of the internship student was different. He was in the last year of an intensification in robotics. He already knew the fundamentals of robotics and and had previously worked with MATLAB and Simulink, but not with LEGO robots. His work had to lead him to the development of computational thinking and advanced programming skills. Therefore, his task was to implement previous research results developed by the research group regarding new path planning techniques. In particular, his goal was to implement the algorithm published in [45], based on clothoidal paths.
- Type C: Research Application
  The third application presented in this study is related to the use of the platform to test new motion planning algorithms developed by the authors’ research group, as the one in [5], where a global path planner is proposed for mobile robots based on the Proper Generalized Decomposition method. In this case, PhD students and researchers had extensive experience programming with MATLAB and Simulink, but not with LEGO robots.

With the aim of assessing the research questions (RQ) stated in Section 3, a set of mini-interviews were planned, where different questions were made to the students in an informal manner and their answers were collected. The questions are detailed in Table 1. In order to assess the learning progress, these questions were made several times along the project: at the beginning (t0), in the middle (t1), and at the end (t2). The questions were slightly modified attending to the specific stage of the project. The answers were rated by the teacher in a Likert scale ranging 1 to 5, from “not at all/very difficult (1)” to “absolutely/very easy (5)”, depending on the answer of the student. Some of the answers to these questions were extracted from the students’ final reports and field notes.

Regarding the external evaluation of the final degree projects (Type A) and the internship project (Type B), Table 2 details the items assessed by the evaluation committee related to the research questions. These items have been extracted from the university evaluation rubric for this type of projects and are rated using a percentage of the accomplishment level (25%, 50%, 75%, 100%). Also, a global evaluation item has been included that indicates the level of knowledge integration.

## 5. Results

The answers to the questions in Table 1 are collected in Table 3. Similarly, the evaluation scores of the items in Table 2 are presented in Table 4.

Regarding the information in Table 1 and Table 3, as every question was made 3 times during the project, an evolution in the learning outcomes is clear from the data. In relation to RQ1, the data show that students develop an increasing understanding of mathematical models as the project goes on, being able to use them in the platform already in the middle of the project (t1), specifically those with previous knowledge in robotics (case B and C). This subjective perception is also validated by the external score given by the evaluation committee to the projects, with a modest result for undergraduate students but higher for the graduate student. This result suggests that having previous mathematical and technical knowledge helps in the consolidation of abstract knowledge and its transference to physical systems.

In relation to RQ2, which assesses the level of procedural competence acquired by students due to the interaction with robots, it can be seen that the students are able to completely build and control the robot in the middle of the project (t1). In this case, no differences have been found in students of different background: neither in the students and the tutor’s perceptions, nor in the external evaluation scores. Additionally, the external score for this RQ is very satisfactory, reaching 100% one of the undergraduate students and the graduate student. This outcome indicates that the interaction with robots using this platform helps students to acquire a high level of procedural competence.

Finally, with respect to RQ3, which evaluates the advantages of the platform in the development of CT skills, the answers of the students indicate an increase in the perception of ease when programming the robot behavior as the project progresses. In this sense, all the students achieved a 100% in item E8 of Table 4, which evaluates the performance of the interaction between the robot and the platform. Also item E7, that accounts for implementation issues, had a good score in two of the three students. These scores suggest that the platform facilitates the development of CT skills in an easy way independently of the students background.

In the following paragraphs, specific and qualitative observations for each project are described. These observations were extracted from the tutors’ opinions.

**Figure 4:**
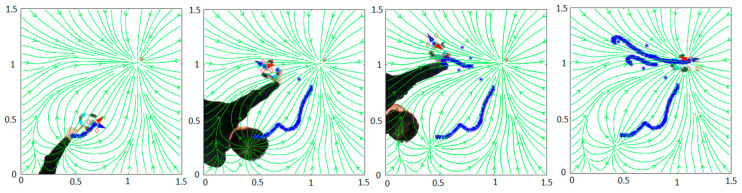
PGD path planner. The red goal is changed in real time.

- Type A: Bachelor’s Degree Final Projects
  The undergraduate students explored the Internet and found open source code developed in MATLAB for typically used robot path planning algorithms. They focused their attention in the website of Rahul Kala, Assistant Professor at the Indian Institute of Information Technology, Allahabad [47]. His website provides open source MATLAB code for some very common planning techniques like for instance A Probabilistic Roadmap (PR), Rapidly-exploring Random Trees (RRT), Genetic Algorithms (GA), Fuzzy Logic (FL), and Artificial Potential Fields (APF). The students downloaded the code and introduced it in the LEGO EV3 robot by means of the Template Simulink block of Figure 1. The students did not have any previous knowledge about path planning algorithms but, even in this case, they were able to successfully implement the interactive applications as a way to apply their knowledge about computational thinking and to understand the principles of robotics.
- Type B: Internship Student Project
  In this case, the student already knew the fundamentals of robotics and motion planning and, for that reason, his task was more challenging than the previous one. In spite of that, he was able to implement the algorithm [45], based on clothoidal paths, and successfully navigate the robot following this type of paths. The difficulty in the implementation was related to the mathematical model of clothoid curves and the linking of several curves to form a path. These curves are not studied in engineering degrees and their use require the development of abstract and computational thinking.
- Type C: Research Application
  In this case, PhD students were able to benefit from the application of complex mathematical and mechanical models at the same time that improved robotics and programming skills, thus developing abstract, procedural, and computational thinking knowledge. The motion planning algorithm described in [46] was implemented in the LEGO EV3 platform and the robot successfully navigated the environment. Figure 4 shows snapshots of the experimental result where the possibility of interaction with the work environment is shown, extracted from a YouTube video [48] of the experimental application.

The complexity of the different interactive projects that the students have been able to develop indicate that the educational platform MATLAB/Simulink/LEGO EV3 offers a lot of advantages with respect to previous versions of LEGO bricks. It is possible to create many different applications in real time to teach any engineering or computer science subject. The computational abilities that can be developed at a low cost together with the possibility of constructing any kind of robot or device (Ballbot, Bicycle, robot arm, etc.) makes the present platform a powerful tool to involve students in a wide variety of subjects at all the educational levels. Moreover, another advantage is that MATLAB and Simulink are running in parallel. While Simulink is a mirror of the code that is running in the LEGO EV3 brick, MATLAB exchanges parameters to control the robot. It makes MATLAB a perfect platform to teach an endlessly amount of algorithms, as artificial vision, teleoperation, etc., since they can be executed in MATLAB without any restriction. The main disadvantage of the platform is that not all the functions that can be used in MATLAB can be compiled and executed in a LEGO robot. For instance, the Fuzzy Logic path planner downloaded from [47] and tested by one of the bachelor students, could not be compiled because it uses the MATLAB fuzzy logic library. A list provided by Mathworks in [49] details the functions that can be used in LEGO bricks.

Programming applications in MATLAB/Simulink to be compiled in C/C++ require programming skills and computational thinking, as well as knowledge of the differences between both programming languages. For example, in C/C++ the variables have to be defined at the beginning, while in MATLAB it is not necessary. Therefore, if further developers download algorithms from the Internet that run in MATLAB, both limitations must be taken into account: not all functions can be compiled on a LEGO robot and it may be necessary to define the variables before compiling in C++.

## 6. Discussion

The new real-time platform has been found to be very useful for developing interactive student projects. In this sense, the projects involve not only programming tasks but interaction with robots that encourage students to integrate the knowledge of the different subjects in the fields of control, robotics, and computer science that they have studied throughout the degree or master.

Patterns have been found in students’ learning that are similar across the different teaching experiments. The results of the study can be summarized as:

- The platforms helps students to understand the purpose and use of mathematical models.
- The platform increases the level of procedural competence acquired by students.
- The platform facilitate the development of CT skills.

A clear limitation of this study is the low number of experiments carried out. Therefore, the conclusions of this work should be considered promising although preliminary. In this sense, it is possible to create many different applications in real time to teach any engineering or computer science subject. The complexity of the different interactive projects that the students have been able to develop indicate that the educational platform MATLAB/Simulink/LEGO EV3 offers a lot of advantages with respect to previous versions of LEGO bricks. The computational abilities that can be developed at a low cost together with the possibility of constructing any kind of robot or device (Ballbot, Bicycle, robot arm, etc.) make the present platform a powerful tool to involve students in a wide variety of subjects at all the educational levels. An advantage of this platform with respect to other approaches is that MATLAB and Simulink are running in parallel. While Simulink is a mirror of the code that is running in the LEGO EV3 brick, MATLAB exchanges parameters to control the robot. It makes MATLAB a perfect platform to teach an endless amount of algorithms, as artificial vision, teleoperation, etc., since they can be executed in MATLAB without any restriction. The main disadvantage of the platform is that not all the functions that can be used in MATLAB, can be compiled and executed in a LEGO robot. For instance, the Fuzzy Logic path planner downloaded from [47] and tested by one of the bachelor students, could not be compiled because it uses the MATLAB fuzzy logic library. A list provided by Mathworks in [49] details the functions that can be used in LEGO bricks. Programming applications in MATLAB/Simulink to be compiled in C/C++ require programming skills and computational thinking, as well as knowledge of the differences between both programming languages. For example, in C/C++ the variables have to be defined at the beginning, while in MATLAB it is not necessary. Therefore, if further developers download algorithms from the Internet that run in MATLAB, both limitations must be taken into account: not all functions can be compiled on a LEGO robot and it may be necessary to define the variables before compiling in C++.

## 7. Conclusions and Further Developments

The present paper validates an educational platform based on the MATLAB-Simulink package with the LEGO EV3 brick for the development of real-time interactive projects with the students as well as for research activities. This kind of projects make it possible for students to integrate abstract, physical, procedural, and logico-mathematical knowledge. The main advantage offered in contrast to previous versions is the possibility to create real-time applications that can interact with the students, which motivates students and lecturers in the learning and teaching task. The present paper tests the platform for some case studies in order to detect the restriction of its applicability. As a drawback, not all MATLAB functions can be used and it is necessary to recode the MATLAB functions to define the variables.

Our future work is focused on the use of this platform in different subjects of the curricula in order to further validate the results of this study. Additionally, the current software platform, based on MATLAB-Simulink, could be extended to other low-cost hardware platforms, such as Arduino and Raspberry Pi, to compare their properties and determine their applicability in different subjects. 

## Figures and Tables

**Table 1:** Questions in the mini-interviews associated with the research questions (RQs).

RQ	ID	Question
1	1	Do you know what mathematical models are used for in robotics?
	2	Do you know what does this matrix represent *?
	3	Do you know how to include it in your project?
	4	Do you know what a Bèzier curve/Clothoid curve/potential field is?
	5	Can you formulate it to include it in your project?
2	6	How easy is to include this part in the robot? (sensor, actuator)
	7	How easy is to connect it?
	8	How easy is to make it work with the platform?
	9	Can you indicate the actuators/sensors/transmission systems in the robot?
	10	Can you identify the control system in the robot?
3	11	How easy is to program a simple motion of the robot?
	12	How easy is to program a kinematic model in the platform?
	13	How easy is to program a complex behavior of the robot?
	14	How easy is to include external code in the platform?
	15	Did the interaction with the robot help you in the development of the program?

* A matrix representing the kinematic model of the robot or the PGD (Proper Generalized Decomposition) approach.

**Table 2:** Evaluation questions for the Type A projects associated to the research questions.

RQ	ID	Item
1	E1	Knowledge of the mathematical models used and implementation
	E2	Understanding of algorithms at theoretical and code level
2	E3	Use of the elements of the robot (sensors, actuators) and connection to the platform
	E4	Knowledge of the control systems used
3	E5	Legibility of the code, kinematic model and behavior
	E6	Temporal efficiency of the code
	E7	Implementation of the external mode (external code) of the platform
	E8	Performance of the interaction between the robot and the platform
Global	E9	Ability to integrate and synthesize the knowledge and skills acquired during the studies evidencing them through the completion of a final degree project

**Table 3:** Answers to the mini-interview questions associated with the research questions.

Project	Time	RQ1					RQ2					RQ3				
		1	2	3	4	5	6	7	8	9	10	11	12	13	14	15
A	t0	1	1	1	1	1	4	4	2	2	2	1	1	1	1	3
	t1	3	2	3	3	3	5	5	4	5	5	3	2	2	2	4
	t2	5	5	4	5	4	5	5	5	5	5	5	4	3	4	5
B	t0	2	3	1	1	1	4	4	4	4	4	3	3	2	2	3
	t1	4	4	4	3	3	5	5	5	5	5	4	4	3	3	4
	t2	5	5	5	5	5	5	5	5	5	5	5	5	5	4	5
C	t0	3	4	2	2	2	4	4	3	3	3	3	4	3	2	4
	t1	4	5	4	4	4	5	5	4	5	5	4	5	4	4	4
	t2	5	5	5	5	5	5	5	5	5	5	5	5	5	5	5

**Table 4:** External evaluation results (in percentage) of Type A and B projects for each item related to each RQ.

Project	RQ1		RQ2		redRQ3				Global
	E1	E2	E3	E4	E5	E6	E7	E8	E9
A1	50%	75%	100%	100%	75%	75%	50%	100%	75%
A2	50%	50%	75%	50%	75%	100%	100%	100%	75%
B	100%	75%	100%	100%	50%	50%	75%	100%	100%

## Data Availability

The data used in this manuscript are included in the text.

## References

[1] Pedersen B.K.M.K., Larsen J.C., Nielsen J., Merdan M., Lepuschitz W., Koppensteiner G., Balogh R., Obdržálek D. (2020). The Effect of Commercially Available Educational Robotics: A Systematic Review. Robotics in Education. RiE 2019, Advances in Intelligent Systems and Computing.

[2] Isabelle M.L.S., Andrade W.L., Lívia M.R.S. Analyzing the Effect of Computational Thinking on Mathematics through Educational Robotics. Proceedings of the IEEE Frontiers in Education Conference (FIE).

[3] Souza I.M.L., Andrade W.L., Sampaio L.M.R., Araujo A.L.S.O. A Systematic Review on the use of LEGO^®^ Robotics in Education. Proceedings of the IEEE Frontiers in Education Conference (FIE).

[4] Danahy E., Wang E., Brockman J., Carberry A., Shapiro B., Rogers C.B. (2014). LEGO-based Robotics in Higher Education: 15 years of Students Creativity. Int. J. Adv. Robot. Syst..

[5] Montés N., Rosillo N., Mora M.C., Hilario L., Lepuschitz W., Merdan M., Koppensteiner G., Balogh R., Obdržálek D. (2019). Real-Time Matlab-Simulink-Lego EV3 Framework for Teaching Robotics Subjects. Robotics in Education. RiE 2018. Advances in Intelligent Systems and Computing.

[6] LEGO (2017). First LEGO League (FLL). http://www.firstLEGOleague.org/.

[7] Weissberger I., Qureshi A. (2014). Delivering Software Engineering Education Through LEGO Robotics. Int. Conf. Comput. Sci. Educ..

[8] Weissberger I., Qureshi A. Evaluating the Effectiveness of LEGO Robots in Engaged Scholarship. Proceedings of the 2015 Annual Global Online Conference on Information and Computer Technology (GOCICT).

[9] Ponce P., Molina A., Hernández L., Acha E., Morales B., Huitron C. (2017). Teaching Math in Elementary Schools by LabVIEW and LEGO Robots. Advances in Automation and Robotic in Latin America. Lecture Notes in Networks and Systems.

[10] Chaudhary V., Agrawal V., Sureka A. An Experimental Study on the Learning Outcome of Teaching Elementary Level Children using LEGO Mindstorms EV3 Robotics Education Kit. Proceedings of the IEEE 8th International Conference on Technology for Education.

[11] Enriquez C., Aguilar O., Dominguez F.D. (2016). Using Robot to Motivate Computational Thinking in High School Students. IEEE Latin Am. Trans..

[12] Llori O., Watchorn A. (2016). Inspiring next generation of engineers through service-learning robotics outreach and mentorship programme. Int. J. Adv. Robot. Syst..

[13] Ozurçon N.Ç., Bicen H. (2017). Does the Inclusion of Robots Affect Engineering Students’ Achievement in Computer Programming Courses?. J. Math.Sci. Technol. Educ..

[14] Kim S.H., Jeon J.W. (2009). Introduction for Freshmen to Embedded Systems Using LEGO Mindstorms. IEEE Trans. Educ..

[15] Perez S.R., Gold-Veerkamp C., Abke J., Borgeest K. A New Didactic Method for Programming in C for Freshmen Students Using LEGO Mindstorms EV3. Proceedings of the IEEE International Conference on Interactive Collaborative Learning.

[16] Zhang J., Irgen-Gioro J. (2016). Teaching Artificial Intelligence Using LEGO. Int. Conf. Front. Educ..

[17] Valera A.A., Soriano A., Vallés M. (2014). Plataformas de bajo coste para la realización de trabajos prácticos de mecatrónica y robótica. Rev. Iberoam. Autom. Inf. Ind..

[18] Cuellar M.P., Pegalajar M.C. (2011). Design and Implementation of Intelligent Systems with LEGO Mindstorm for Undergraduate Computer Engineers. Comput. Appl. Eng. Educ..

[19] Pinto M., Moreira A.P., Matos A. (2012). Localization of mobile robots using an extended Kalman filter in a LEGO NXT. IEEE Trans. Educ..

[20] Poikselka K., Vallivaara L.I., Roning J. Evolutionary robotics on LEGO NXT Platform. Proceedings of the IEEE International Conference on Tools with Artificial Intelligence.

[21] Toivonen T., Jormanainen I., Tukiainen M. (2017). An Open Robotics Environment Motivates Students to Learn the Key Concepts of Artificial Neural Networks and Reinforcement Learning. International Conference on Robotics and Education.

[22] Zaldivar D., Cuevas E., Maciel O., Valdivia A., Chavolla E., Oliva D. (2019). Learning classical and metaheuristics optimization techniques by using educational platform based on LEGO robots. Int. J. Electr. Eng. Educ..

[23] Bradley P.J., Juan A., Zamorano J., Brosnan D. (2012). Platform for Real Time Control Education with LEGO MINDSTORM. IFAC Proc. Vol..

[24] Rodriguez C., Guzman J.L., Berenguel M., Dormido S. (2016). Teaching real-time programming using mobile robots. IFAC Symp. Adv. Control Educ..

[25] Jovic N.D., Matijevic M.S. LEGO Web laboratory at University of Kragujevac. Proceedings of the Global Engineering Education Conference.

[26] Gomez-de-Gabriel J.M., Mandow A., Fernandez-Lozano J., Garcia-Cerezo A.J. (2011). Using LEGO NXT Mobile robots with LABVIEW for undergraduate Courses on Mechatronics. IEEE Trans. Educ..

[27] Gómez-de-Gabriel J.M., Mandow A., Fernandez-Lozano J., Garcia-Cerezo A. (2015). Mobile Robot Lab Project to Introduce Engineering Students to Fault Diagnosis in Mechatronic Systems. IEEE Trams. Educ..

[28] Kim Y. (2011). Control system lab using a LEGO Mindstorms NXT Motor System. IEEE Trams. Educ..

[29] Cruz-Martín A., Fernández-Madrigal J.A., Galindo C., González-Jiménez J., Stockmans-Daou C., Blanco-Claraco J.L. (2012). A LEGO Mindstorms NXT approach for teaching at data acquisition, control systems engineering and real-time systems undergraduate courses. Comput. Educ..

[30] Canale M., Casale-Brunet S. (2014). A multidisciplinary approach for Model Predictive Control Education: A LEGO Mindstorms NXT-based framework. Int. J. Control Autom. Syst..

[31] Kim S., Oh H., Choi J., Tsourdos A. (2014). Using Hands-on Project with LEGO Mindstorms in a Graduate Course. Int. J. Eng. Educ..

[32] García-García R.A., Arias-Montiel M. (2016). Linear Controllers for the NXT Ballbot with Parameter Variations Using Linear Matrix Inequalities. IEEE Control Syst..

[33] Savu D., Sandru L.A., Crainic M.F., Moldovan C., Dolga V., Preitl S. (2016). Multiple Methods of Data Acquisition for a LEGO NXT 2 Mobile Robot: The use of a second NXT 2 Hardware Platform. Proceedings of the 5th International Conference on Mechatronics and Control in Engineering.

[34] Ding J., Li Z., Pan T. (2017). Control System Teaching and Experiment Using LEGO MINDSTORMS NXT Robot. Int. J. Inf. Educ. Technol..

[35] Serrano V., Thompson M., Tsakalis K. (2017). Learning Multivariable controller design: A Hands-on Approach with LEGO Robotic Arm. Advances in Automation and Robotic in Latin America. Lecture Notes in Networks and Systems.

[36] Indri M., Lazzero I., Bona B. Robotics education: Proposals for laboratory practices about manipulators. Proceedings of the IEEE International Conference on Emerging Technologies and Factory Automation.

[37] Basso M., Innocenti G. (2015). LEGO-Bike: A Challenging Robotic Lab Project to Illustrate Rapid Prototyping in the Mindstorms/Simulink Integrated Platform. J. Comput. Appl. Eng. Educ..

[38] Vokorokos L., Mihalov J., Chovancova E. (2015). Potential of LEGO EV3 Mobile robots. Acta Electrotech. Inf..

[39] Guidi G., Maffei N., Ciarmatori A., Mistretta M.G., Gottardi G., Costi T., Guidi G., Maffei N., Vecchi C., Baldazzi G. (2015). Real-time lung tumour motion modelling for adaptative radiation therapy using LEGO mindstorms. J. Mech. Med. Biol..

[40] Trinath B.P., Mija S.L. Balancing of Two Wheeled Inverted Pendulum using SOSMC and Validation on LEGO EV3. Proceedings of the IEEE International Conference on Power Electronics, Intelligent Control and Energy Systems.

[41] Al-Halhouli A.A., Qitouqa H., Malkosh N., Shubbak A., Al-Gharabli S., Hamad E. (2016). LEGO Mindstorms NXT for elderly and visually impaired people in need: A platform. Technol. Health Cares.

[42] Maharuddin M.F., AbdulGhani N.M., Jamin N.F. (2018). Two-wheeled LEGO EV3 Robot Stabilisation Control Using Fussy Logic Based PSO Algorithm. J. Telecomun. Electr. Comput. Eng..

[43] Bevrnja F., Bevrnja M., Petrovic M. (2019). Dynamic analysis, modelling and control of the LEGO EV3 modular mobile platform. Period. Eng. Nat. Sci..

[44] Blumenfeld P.C., Soloway E., Marx R.W., Krajcik J.S., Guzdial M. (1991). Palincsar, Motivating project-based learning: Sustaining the doing, supporting the learning. Educ. Psychol..

[45] Montés N., Mora M.C., Tornero J. Trajectory generation based on Rational Bezier Curves as clothoids. Proceedings of the IEEE International Conference on Robotics and Automation.

[46] Montés N., Chinesta F., Falcó A., Mora M.C., Hilario L., Duval J. A PGD-based framework for robot global path planning: A primer. Proceedings of the 16th International Conference on Informatics in Control, Automation and Robotics (ICINCO 2019).

[47] Khala R. Code for robot path planning. http://www.rkala.in/codes.php.

[48] Montés N., Chinesta F., Mora M.C., Falcó A., Hilario L. (2020). PGD-Based Framework for Potential-Guided Robot Path Planning. www.youtube.com/watch?v=LC_kFZPmOH0.

[49] (2020). Mathworks. http://www.mathworks.com/help/coder/ug/functions-and-objects-supported-for-cc-code-generation.

